# Supplementary material for: Effects of a choral program combining wind instrument performance and breathing training on respiratory function, stress, and quality of life in adolescents: A randomized controlled trial
Source: PLoS One. 2024 May 7;19(5):e0276568. doi: 10.1371/journal.pone.0276568 (PMC11075825; doi:10.1371/journal.pone.0276568)
Supplement: S1 File — (PDF) [file pone.0276568.s002.pdf]

기관생명윤리위원회 심의결과 안내서

1. 연구과제 개요

과제관리번호	1040647-201910-HR-007-02					
연구과제명	합창과 호흡훈련을 병행한 복합예술교육프로그램이 청소년의 호흡기능, 스트레스 그리고 삶의 질에 미치는 영향					
연구책임자	성명	김지연	소속	대전대학교 방송공연예술학과		
연구기간	윤리위원회 승인일 ~ 2020년 6월 30일					
심의신청일	2019년 10월 17일	심의일	2019년 10월 24일	결과확정일	2019년 12월 11일	
심의종류	( ) 심의면제					
	( ) 재심의					
	( ) 정규심의 (●) 신속심의					
	( ) 지속심의 ※ **심의 주기 : ( ) 3개월 ( ) 6개월 ( ) 12개월 ( ) 기타 : ______**					
연구정보	목적	(●) 학술용	( ) 기타 : ______			
	종류	(●) 인간대상연구	(●) 실험군-대조군 연구	( ) 코호트 연구	( ) 기타:	
			( ) 단면조사연구	( ) 설문조사		
			( ) 행동관찰	( ) 면담조사		
연 구 비	지원기관	한국연구재단	연구비	15,000,000원		

2. 심의 내용

연구계획서

- 정규심의 후 보완된 내용에 대한 재심의로 전 심의결과에서 지적된 미성년자의 보호자 동의, 이상반응 조치사항, 연구자의 안전성, 용어의 설명, 모집공고문 등을 수정하여 제출함. 제출된 내용이 잘 보완기술되어 생명연구윤리에 부합하다고 판단됨.

연구대상자 안전/ 개인정보 보호 대책 확인

- 수정된 계획서 내용에 자료의 처리와 보관에 대해 잘 명시되어 있어 대상자의 개인정보 보호에 대한 대책도 잘 수립되어 있음.

결과

- 위의 사항을 종합하여 이 연구계획서는 **승인**으로 판단함.

3. 심의 결과

심의 유형	신속심의
심의 결과	승인
승인 번호	1040647-201910-HR-007-03
유효 기간	2019년 12월 11일 - 2020년 12월 10일<br>• 총 신청 연구기간이 IRB 연구승인 유효기간을 초과할 경우, 유효기간 만료 이전에 '지속심사' 승인을 받아야 연구지속 진행이 가능합니다.
심의 결과 후 과정	• 승인 연구의 경우는 연구계획서대로 연구를 진행하시면 됩니다. 연구 종료시 연구 결과보고서(서식19, 서식20)를 제출하십시오. 연구 중 변경 사항이 있는 경우에는 재심의 신청하십시오.<br>• 조건부 승인, 보완후 재심의인 경우 수정사항을 수정한 후 연구계획서를 2주 내에 다시 제출하십시오.<br>• 동일 연구계획서에 대하여 2회 이상의 재심은 하지 않습니다. 2회 재심을 받은 경우 신규연구계획서로 다시 제출하여야 합니다.<br>• 심의 결과에 이의가 있을 경우 통보일로부터 2주일 이내에 이의 신청[서식28]을 할 수 있습니다.

귀하가 요청한 연구 계획의 심의 결과를 위와 같이 안내합니다.

2019년 12월 11일

대전대학교 기관생명윤리위원회 위원장 (인)

No. 승인
2019. 12. 11
대전대IRB

2019 복합예술 교육 프로그램

연구대상 모집

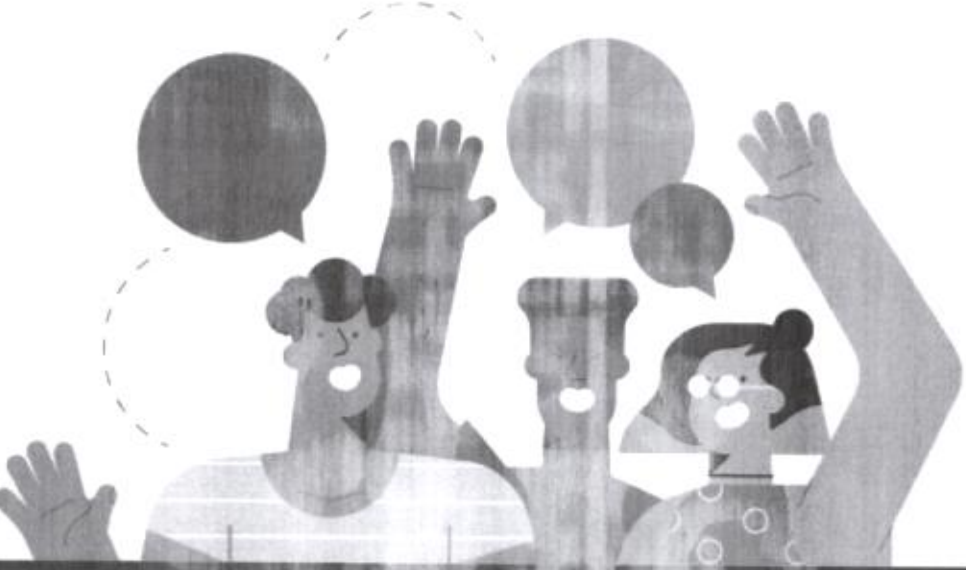

연구과제

합창과 호흡 훈련을 병행한
복합예술교육프로그램이
청소년의 호흡기능, 스트레스
그리고 삶의 질에 미치는 영향

모집인원

선착순 60명

모집대상

□만14세 이상 만 24세 미만 지역사회 중.고.대학생청소년
□호흡계관련 만성질환을 가지고 있지 않은 자
□합창 및 악기연주에 정형외과적 문제가 없는 자
□본연구를 이해하고 참여를 희망하는 자
□미성년자의 경우 보호자의 동의가 있는 자

모집기간

2019.12.15~2019.12.30

이메일: jymusic@dju.kr
담당자 전화:010-4816-6929

No. 승인
2019. 12. 11
대전대IRB

연구 대상자 동의 설명서

제목: 합창과 호흡훈련을 병행한 복합예술교육프로그램이 청소년의 호흡기능, 스트레스, 삶의 질에 미치는 영향

연구책임자	김 지 연	소속	대전대학교 방송공연예술학과	직책	조교수
연구담당자	김 병 수	소속	대전대학교 물리치료학과	직책	석사생

귀하께서는 본 연구에 참여하시도록 제안 받았습니다. 이 연구는 참여자의 권리를 보호할 책임이 있는 대전대학교 기관생명윤리위원회 (IRB)의 승인을 받은 연구입니다.

아래의 내용은 이번 연구의 목적과 귀하의 권리 및 역할 등에 대하여 설명 드리고자 마련된 것입니다. 귀하가 연구 참여 동의 전에 이 동의서를 읽고 이해하는 것이 중요합니다. 시간을 가지고 충분히 심사숙고 하시어 읽으신 후, 원하신다면 가족이나 다른 사람과 상의하셔도 좋고, 또 궁금하신 사항이 있으시면 연구책임자나 다른 연구담당자에게 질문하셔도 좋습니다.

1. 연구의 목적(이 연구는 왜 실시합니까?)

본 연구의 목적은 합창과 호흡훈련을 병행한 복합예술교육프로그램이 지역사회 청소년의 호흡기능, 스트레스 그리고 삶의 질에 미치는 영향을 조사하기 위함입니다.

※ 호흡기능 : 산소 공급력에 관계하는 폐의 환기 능력 및 가스교환에 관련하는 여러 조건, 즉 폐 내의 가스 분포, 폐포닥의 성상 및 폐순환 등과 체순환을 포함한 기능과 호흡과 관련된 근육의 근력정도를 말합니다.

※ 삶의 질 : 개인이 삶을 살아갈 때 만족감, 행복감 등의 주관적인 삶의 만족 정도를 의미합니다.

2. 연구대상자 선정 기준

선정기준	(1) 지역사회 거주 만 14세 이상 24세 미만인 중 고 대학생인 자<br>(2) 호흡기 관련 만성질환을 가지고 있지 않은 자(감기제외)<br>(3) 합창 및 악기연주에 정형외과적 문제가 없는 자<br>(4) 본연구의 목적을 이해하고 참여에 동의한 자<br>(5) 만 19세 미만 미성년자의 경우 부모의 동의를 받은자
제외기준	(1) 항 정신성 약물을 복용하고 있는 자<br>(2) 심한 만성호흡기질환으로 평가 및 실험 참여가 어려운 자(감기제외)<br>(3) 본연구의 모든 절차수행시 통증 또는 불편감으로 참여가 불가능한 자<br>(4) 의사소통에 문제가 있는 자

3. 연구대상자 정보 및 수

지역사회 거주 만 14세 이상 24세 미만인 중,고,대학생인 자 120명이 참여합니다.

4. 연구대상자 연구 참여 예정기간 및 참여 시간.

연구참여 기간은 총 3개월이며 주 1회 2시간/회입니다.

5. 연구 절차(만일 연구에 참여하면 어떤 과정이 진행 됩니까?)

본 연구는 합창과 호흡훈련을 병행한 예술교육프로그램으로 구성된 복합예술교육훈련프로그램 중재 연구 이며, 연구참여 기간중 첫주차와 프로그램이 끝난 주차에 평가가 진행될 예정입니다.

평가는 호흡기능을 측정할수 있는 폐기능 검사와 호흡근력검사, 스트레스를 평가할 수 있는 사회심리적 스트레스수준검사, 삶의 질을 평가하는 한국판 세계보건기구 삶의 질 간편형 척도로 검사하여 실험 전후 값을 비교할 예정입니다.

6. 연구 동의 후 철회(참여 도중 그만두어도 됩니까?)

본 연구에 참여하는 과정에서 평가 혹은 중재 과정동안 어떠한 불편감 또는 불쾌감으로 인하여 연구 참여를 그만두고 싶을 경우, 언제든지 그만두어도 됩니다.

7. 연구에 참여함으로써 예상되는 위험성이나 불편 사항(부작용이나 위험요소는 없습니까?)

호흡기능을 평가하는 과정에서 최대호기와 최대흡기를 반복해야 하는 경우 호흡곤란 또는 어지러움이 발생 할 수 있습니다. 하지만, 휴식으로 충분히 회복될 수 있으며, 이를 지속적으로 모니터링하기 위해 혈중 산소 포화도 수치와 혈압을 측정 할 예정입니다. 이로 인한 불편감에 의해 연구 참여를 그만두고 싶을 경우 연구 참여를 즉시 그만 둘 수 있습니다.

8. 연구와 관련된 피해 발생 시 보상

연구 계획서의 절차와 관련하여 이상반응 또는 기타 다른 문제가 발생할 경우 대상자는 즉시 평가자에게 문의해야 하며, 연구자는 최선의 의학적 조치를 취할 것입니다. 또한 피해자 보상규약에 따라 보상할 것입니다. 다만 연구와 인과 관계가 없는 사건이나 사고에 대해서는 보상하지 않습니다.

9. 귀하의 인적사항에 대한 비밀보장(연구에서 얻은 모든 개인 정보의 비밀은 보장됩니까?)

연구 중 취득한 모든 정보는 비밀이 보장되고 보안을 위해 귀하의 신원을 확인 할 수 없도록 식별코드로 관리하며, 연구 목적 이외에는 사용하지 않습니다. 연구 결과가 출판된 경우라도 귀하의 신상정보는 비밀상태로 유지됩니다.

10. 연구 대상자에 제공되는 편의(이 연구에 참여시 참여자에게 이득이 있습니까?)

본 연구에 참여하는 대상자들은 합창과 호흡훈련을 병행한 예술교육프로그램으로써 합창과 호흡훈련을 통한 스트레스 감소, 삶의 질 및 호흡기능 증진 훈련을 제공받을 수 있습니다. 또한 본인의 호흡기능 및 스트레스, 삶의 질 수준을 알 수 있습니다.

11. 연구 대상자 연구 불참시 불이익(만일 이 연구에 참여하지 않는다면 불이익이 있습니까?)

연구에 참여하지 않더라도 불이익은 없습니다.

12. 연구 참여 댓가 지급 여부 (이 연구에 참가하면 댓가가 지급됩니까?)

프로그램에 참여하여 제공받는 중재와 평가에 대한 댓가는 발생하지 않습니다. 다만 훈련프로그램은 무상으로 제공할 것이며, 프로그램에 사용되는 관리품을 무상으로 관리할 예정입니다.

13. 각 연구 관련 / 책임자(연구에 대한 문의는 어떻게 합니까?)

※ 본 연구에 대해 질문이 있거나 연구 중간에 문제가 생길 시 다음 연구 담당자에게 연락하십시오.

연구책임자	김 지 연	소속	대전대학교<br>방송공연예술학과	직책	조교수	H.P	010-4816-6929
연구담당자	김 병 수	소속	대전대학교<br>물리치료학과	직책	석사생	H.P	010-7192-8973

※ 만일 어느 때라도 연구대상자로서 귀하의 권리에 대한 질문이 있다면 다음의 대전대학교 기관생명윤리위원회에 연락하십시오.

대전대학교 기관생명윤리위원회 전화번호 : 042)280-2029

13 14 15 16 17 18 19 20 21 22 23 24 1 2 3
No. 승인
2019. 12. 11

No. 승인
2019. 12. 11
대전대IRB

동 의 서

제목: 합창과 호흡훈련을 병행한 복합예술교육프로그램이 청소년의 호흡기능, 스트레스, 삶의 질에 미치는 영향

1. 나는 이 설명을 읽었으며 담당 조사원과 이에 대하여 의논하였습니다.

2. 나는 위험과 이득에 관하여 들었으며 나의 질문에 만족할 만한 답변을 얻었습니다.

3. 나는 이 연구에 참여하는 것에 대하여 자발적으로 동의합니다.

4. 나는 나의 사진 및 동영상 자료를 촬영하는 것에 대하여 자발적으로 동의합니다.

5. 나는 이 구술에서 제공된 나에 대한 정보나 관련 사진 및 동영상 자료를 현행 법률과 대전대학교 기관생명윤리위원회 규정이 허용하는 범위 내에서 연구자가 수집하고 처리하는 데 동의합니다.

6. 나는 담당 연구자나 위임받은 대리인이 연구를 진행하거나 결과 관리를 하는 경우와 학교 당국 및 대전대학교 기관생명윤리위원회가 실태 조사를 하는 경우에는 비밀로 유지되는 나의 개인 신상 정보를 열람하는 것에 동의합니다.

7. 나는 언제라도 이 사업에의 참여를 철회할 수 있고 이러한 결정이 나에게 어떠한 해도 되지 않을 것이라는 것을 압니다.

8. 나의 서명은 이 동의서의 사본을 받았다는 것을 뜻하며 사업 참여가 끝날 때까지 사본을 보관하겠습니다.

[구술자]	성명 : ________	서명 : ____________	날짜 : 년 월 일
[동의받은 조사원 성명]	성명 : ________	서명 : ____________	날짜 : 년 월 일
[연구책임자]	성명 : ________	서명 : ____________	날짜 : 년 월 일

※ 해당되는 경우

[법적 대리인]	성명 : ________	서명 : ____________	날짜 : 년 월 일
[입회인]	성명 : ________	서명 : ____________	날짜 : 년 월 일

승인
No.
2019. 12. 11
대전대IRB

삶의 질 측정검사(WHOQOL-K)

아래의 실문은 지난 2주 동안(오늘을 포함해서) 전반적인 삶에 대해 묻는 질문입니다. 최근 귀하의 삶에 대해 잘 표현하는 문항에 √로 표시하여 주십시오

번호	문항내용	매우 나쁨	나쁨	그저 그렇다	좋음	매우 좋음
1	당신의 삶의 질을 어떻게 평가하시겠습니까?	①	②	③	④	⑤
번호		매우 불만족	불만족	그저 그렇다	만족	매우 만족
2	당신의 건강상태에 대해 얼마나 만족하고 있습니까?	①	②	③	④	⑤
번호	문항내용	전혀 아니다	약간 그렇다	그렇다	많이 그렇다	매우 그렇다
3	신체적 통증으로 인해 내가 해야 할 일을 어느 정도 방해를 받는다고 느낍니까?	①	②	③	④	⑤
4	일상생활을 잘하기 위해서 치료가 필요합니까?	①	②	③	④	⑤
5	당신은 인생을 즐기고 있습니까?	①	②	③	④	⑤
6	당신의 삶이 어느 정도 의미 있다고 느낍니까?	①	②	③	④	⑤
7	당신은 정신을 잘 집중할 수 있습니까?	①	②	③	④	⑤
8	당신은 일상생활에서 안전하다고 느낍니까?	①	②	③	④	⑤
9	건강에 좋은 주거환경(공해, 기후, 소음, 쾌적함)에 살고 있습니까?	①	②	③	④	⑤
번호	문항내용	전혀 아니다	약간 그렇다	그렇다	대부분 그렇다	항상 그렇다
10	일상생활을 위한 충분한 에너지(기력)가 있습니까?	①	②	③	④	⑤
11	당신의 신체적 외모에 만족합니까?	①	②	③	④	⑤
12	당신은 당신의 필요를 만족시킬 수 있는 충분한 돈을 가지고 있습니까?	①	②	③	④	⑤
13	당신은 매일 매일의 삶에서 당신이 필요로 하는 정보를 쉽게 구할 수 있습니까?	①	②	③	④	⑤
14	당신은 제시(여가)활동을 위한 기회를 가지고 있습니까?	①	②	③	④	⑤
15	당신은 얼마나 잘 돌아다닐 수 있습니까?	①	②	③	④	⑤
번호	문항내용	매우 불만족	불만족	보통	만족	매우 만족
16	당신은 당신의 수면에 대해 만족하고 있습니까?	①	②	③	④	⑤
17	당신은 일상생활의 활동을 수행하는 당신의 능력에 만족하고 있습니까?	①	②	③	④	⑤
18	당신은 당신의 일할수 있는 능력에 대해 만족하고 있습니까?	①	②	③	④	⑤
19	당신은 당신 스스로에게 만족하고 있습니까?	①	②	③	④	⑤
20	당신은 당신의 개인적 대인관계에 대해 만족하고 있습니까?	①	②	③	④	⑤
21	당신은 당신의 성생활에 대해 만족하고 있습니까?	①	②	③	④	⑤
22	당신은 당신의 친구로부터 받고있는 도움에 대해 만족하고 있습니까?	①	②	③	④	⑤
23	당신은 당신이 살고 있는 장소의 상태에 대해 만족하고 있습니까?	①	②	③	④	⑤
24	당신은 의료서비스를 쉽게 받을 수 있다는 점에 만족하고 있습니까?	①	②	③	④	⑤
25	당신은 당신이 사용하고 있는 교통수단에 대해 만족하고 있습니까?	①	②	③	④	⑤
번호	문항내용	전혀 아니다	드물게 그렇다	제법 그렇다	자주 그렇다	항상 그렇다
26	당신은 침울한 기분, 절망, 불안, 우울감과 같은 부정적인 감정을 얼마나 자주 느낍니까?	①	②	③	④	⑤

No. 승인
2019. 12. 11
대전대IRB

사회 심리적 건강(PWI-SF)

※ 아래의 질문은 최근 몇 주 동안에 경험하셨거나 느끼셨던 육체적 심리적 상태에 대해 물어보는 것입니다. 해당되는 곳에 √표하여 주십시오.

항 목	항상 그렇다	대부분 그렇다	약간 그렇다	전혀 그렇지 않다
1. 현재 매우 편안하며 건강하다고 느낀다				
2. 잠자고 난 후에도 개운한 감이 없다.				
3. 매우 피곤하고 지쳐 있어 먹는 것조차도 힘들다고 느낀다.				
4. 근심걱정 때문에 편안하게 잠을 자지 못한다				
5. 정신이 맑고 깨끗하다고 느낀다.				
6. 기력(원기)이 왕성함을 느낀다.				
7. 밤이면 심란해지거나 불안해 진다.				
8. 대다수의 사람들과 마찬가지로 나를 잘 관리해 나간다고 생각한다.				
9. 전체적으로 현재 내가 하고 있는 일은 잘되어가고 있다고 느낀다.				
10. 내가 행한 일의 방법이나 절차에 만족한다.				
11. 어떤 일을 바로 착수(시작)할 수 있다				
12. 정상적인 일상생활을 즐길 수 있다				
13. 안절부절 못하거나 성질이 심술궂게 되어진다.				
14. 나에게 닥친 문제를 해결해 나갈 수 있다.				
15. 불행하고 우울함을 느낀다				
16. 나 자신에 대해 신뢰감이 없어지고 있다				
17. 모든 것을 고려해 볼 때 행복감을 느낀다				
18. 삶을 살아갈 만한 가치가 있다고 느낀다				

No. 승인
2019. 12. 11
대전대IRB

평 가 지

이름		연령		성별	
Height(cm)		Weight(kg)		BMI(kg/m^2)	

1. 귀하께선 현재 알고 있는 심혈관/호흡계통 질환을 가지고 계신가요?

2. 귀하께서 알고 있는 만성 근골격계 질환이 있으신가요?
(만성 근골격계질환이란 만성적으로 알고 있는 뼈대 및 근육이상증상입니다.)

3. 복용하고 계신 약물이 있으신가요?

4. 최근 3개월간 병원에 입원하신적이 있으신가요? 있다면 어떤 이유인지 서술해주시길 바랍니다.

5. 활력징후검사

최대 수축기혈압		안정시 혈압	
심박수		SpO_2(산소포화도)	

No. [illegible]
2019. 12. 11
대전대IRB

폐기능 검사

호흡근력측정

	1st	2ed	3rd
MIP			
MEP			

결과지 부착
