# Supplementary material for: Effects of a choral program combining wind instrument performance and breathing training on respiratory function, stress, and quality of life in adolescents: A randomized controlled trial
Source: PLoS One. 2024 May 7;19(5):e0276568. doi: 10.1371/journal.pone.0276568 (PMC11075825; doi:10.1371/journal.pone.0276568)
Supplement: S2 File — (PDF) [file pone.0276568.s003.pdf]

Notification of Review Results of Institutional Life Ethics Committee

1. Summary of Research Proposal

Authorization Number	1040647-201910-HR-007-03		
Name of Research Proposal	The Effects of a Combined Arts Education Program Combined with Choral and Breathing Training on Adolescents' Respiratory Function, Stress and Quality of Life		
Principal Investigator	Ji-Yeon Kim	Institute Name	Dept. of Broadcating and performing Arts. School. Daejeon University
Application Date	10. 17. 2019	Review Date	12. 11. 2019
Review Results	approval		
Expiry Date	12. 11. 2019 ~ 12. 10. 2020		
	• If the total research period exceeds the expiry date of IRB approval of the research period, you should receive the approval of 'continuing review" before the expiry date to continue the research project.		

This is to notify that Daejeon University Institutional Life Ethics committee

has examined your research proposal and made the decision as described above.

2019year 12month 11day

The Chairperson of Daejeon University Institutional

Life Ethics committee (sign)
