# Supplementary material for: Effects of a choral program combining wind instrument performance and breathing training on respiratory function, stress, and quality of life in adolescents: A randomized controlled trial
Source: PLoS One. 2024 May 7;19(5):e0276568. doi: 10.1371/journal.pone.0276568 (PMC11075825; doi:10.1371/journal.pone.0276568)
Supplement: Attachment — Submitted filename: Response to Reviewers.docx [file pone.0276568.s004.docx]

**PONE-D-22-27071**

**Effects of a choral program combining wind instrument performance and breathing training on respiratory function, stress, and quality of life in adolescents: A randomized controlled trial**

**PLOS ONE**

Before responding to the review comments, thank you for your interest in our study.

***Reviewer 1***

A: The novelty and clinical purpose of this study is to improve stress by providing various music programs to adolescents with stress caused by various factors in modern society. It is also considered important to improve their well-being by improving not only the psychological aspects, but also the physical functions (respiratory function in this study). These efforts are thought to be able to suggest meaningful interventions not only for clinical respiratory function, but also for patients with mental stress.

**Q1. Abstract:**

- you should write subtitle for each part of Abstract

- The background needs to be shortened.

- Methods section is poorly framed. It has to be re-written.

- Demographic profile of patients is not mentioned.

**Answer.** Modifications have been completed according to your request.

**Q2. Method**

-what is the Type of randomization; details of any restriction (such as blocking and block size)

-what is the Mechanism used to implement the random allocation sequence (such as sequentially numbered containers), describing any steps taken to conceal the sequence until interventions were assigned

**Answer.** This study proceeded with random order and group assignment through the coding of the R Studio program. Corrected the content accordingly.

-Who generated the random allocation sequence, who enrolled participants, and who assigned participants to interventions

**Answer.** The person who conducted the randomization was only one person in charge of the research, and these contents were modified and added to the text.

-Blinding If done, who was blinded after assignment to interventions (for example, participants, care providers, those assessing outcomes) and how

**Answer.** The subject did not know what intervention would be assigned, and this information was added to the text.

-Participants were warmed up and cool down using breathing exercises for 10 min before and after the program started (which types of breathing and how the participant did that and why you select that types)

**Answer.** Modification was completed by adding contents related to breathing training.

**Q3. Discussion**

The discussion section needs to be described scientifically. Kindly frame it along the following lines:

1-Main findings of the present study

2-Comparison with other studies

2-Implication and explanation of findings

**Answer.** The contents of the overall review were revised and described in the main text. However, there are no papers to compare with this study. This thesis is the first study to provide various music programs to community youth. This paper was provided to prove the results of this study, rather than a discussion in the form of contrasting them. I would appreciate it if the reviewer would spare no advice after reviewing it again.

**Q4. Other review opinions**

**Answer.** The rest of the context and formatting were additionally modified. This concludes reviewer 1's opinion. Thank you for your interest in my research. May God's grace always be with you

***Reviewer 2***

**Q1.** The term << breathing training>> in the title doesn't explore the act intervention in the study, as it was done through the two method of intervention not in separated intervention. The author doesn't mention body mass index as one of the inclusion criteria for the selected participants. Small sample size as sample size calculation wasn't done.

**Answer.** In this study, breathing training was provided to G1 and G2, and since it was compared with the control group, it was decided to keep the format as it is. I would really appreciate it if you could give me some careful advice in this regard. The fact that no sample size calculations were performed is noted in Limitations.

***Reviewer 3***

**Q1. Abstract:**

It is better to formulate abstract in a structured manner

Methods section is poorly framed. It has to be re-written.

Demographic profile of patients is not mentioned.

Briefly mention your study findings

Add key words

**Answer.** The abstract was revised based on the review comments above.

**Q2. Introduction**

Explain the rationale of the study. Kindly focus on three elements of introduction.

a. What is known about the topic? (Background)

b. What is not known? (The research problem)

c. Why the study was done? (Justification)

**Answer.** This researcher believes that it is formatted as advised by the reviewer. If it is not enough, I would appreciate it if you could give me some careful advice again.

**Q3. Methods**

How was sample size determined?

It is better to name groups by intervention techniques,not experimental group

What was the method used to generate the random allocation sequence?

Explain the type of randomization.

Was there any restriction like blocking and block size?

What kind of mechanism was used to implement the random allocation sequence?

Were any steps taken to conceal the sequence until interventions were assigned?

Who generated the random allocation sequence?

Who enrolled participants?

Who assigned participants to interventions?

What methods were used for analysis of drop out cases

**Answer.** Since the above content was the same review opinion as Reviewer1, the correction was completed in combination with the items Reviewer3 said. Thank you very much for your thoughtful review comments.

**Q4. The other review opinions**

a. Results need to provide answers to the questions raised/researchable problem

b. Results need to follow ABC (accuracy, brevity, clarity)

The discussion section needs to be described scientifically. Kindly frame it along the following lines:

i. Main findings of the present study

ii. Comparison with other studies

iii. Implication and explanation of findings

iv. Strengths and limitations

v. Conclusion, recommendation and future direction.

2. Explain in short about trial limitations.

3. Describe sources of potential bias and imprecision

**Answer.** The contents of the overall review were revised and described in the main text. However, there are no papers to compare with this study. This thesis is the first study to provide various music programs to community youth. This paper was provided to prove the results of this study, rather than a discussion in the form of contrasting them. I would appreciate it if the reviewer would spare no advice after reviewing it again.

**With the above, we will send the responses to the reviewers' review opinions of this researcher.**

**I would like to thank this researcher for helping me to publish a better study, and I would appreciate it if you would always give me advice if there are any deficiencies in corrections.**

**P.s. God bless you always**
