# Supplementary material for: Effects of a choral program combining wind instrument performance and breathing training on respiratory function, stress, and quality of life in adolescents: A randomized controlled trial
Source: PLoS One. 2024 May 7;19(5):e0276568. doi: 10.1371/journal.pone.0276568 (PMC11075825; doi:10.1371/journal.pone.0276568)
Supplement: Attachment — Submitted filename: Response to Reviewers (2nd).docx [file pone.0276568.s005.docx]

**PONE-D-22-27071R1**

**Effects of a choral program combining wind instrument performance and breathing training on respiratory function, stress, and quality of life in adolescents: A randomized controlled trial**

**PLOS ONE**

Before responding to the review comments, thank you for your interest in our study.

***Reviewer 1***

Thanks a lot for your help in the revision of my thesis. May God bless you always...

***Reviewer 2***

Thanks a lot for your help in the revision of my thesis. May God bless you always...

***Reviewer 4***

**<Major revisions>**

1) Tables 2 and 3: If the interaction effects are significant, provide an interpretation of these results, but do not test main effects because the tests for main effects are uninteresting in light of significant interactions. If interaction effects are non-significant, drop the interaction effects from the model and test the main effects. Determining which results to present when testing interactions is often a multi-step process.

A: We have accepted your request and completed the correction.

2) Tables 2 and 3: Provide p-values instead of t- and F-values.

A: We have accepted your request and completed the correction.

3) Provide meaningful interpretations of significant interaction effects.

A: Dear reviewer, I think that the meaning of interaction has been sufficiently provided in the discussion. If there are any deficiencies, we will proceed with additional corrections later. Thanks a lot.

**<Minor revisions>**

1) Indicate the date range subjects were enrolled in the study.

A: We have accepted your request and completed the correction.

2) Line 101: If block randomization was used, indicate the block size.

A: We have accepted your request and completed the correction.

3) State and justify the study’s target sample size with a pre-study statistical power calculation. The power calculation should include: (1) the estimated outcomes in each group; (2) the α (type I) error level; (3) the statistical power (or the β (type II) error level); (4) the target sample size and (5) for continuous outcomes, the standard deviation of the measurements.

A: We have accepted your request and completed the correction.

4) Table 1:

- In addition to the frequency, provide the percent female.

- In the last column, provide p-values instead of F/chi-square.

A: We have accepted your request and completed the correction.

**With the above, we will send the responses to the reviewers' review opinions of this researcher.**

**I would like to thank this researcher for helping me to publish a better study, and I would appreciate it if you would always give me advice if there are any deficiencies in corrections.**

**P.s. God bless you always**
