# Supplementary material for: Effects of a choral program combining wind instrument performance and breathing training on respiratory function, stress, and quality of life in adolescents: A randomized controlled trial
Source: PLoS One. 2024 May 7;19(5):e0276568. doi: 10.1371/journal.pone.0276568 (PMC11075825; doi:10.1371/journal.pone.0276568)
Supplement: Attachment — Submitted filename: Response to Reviewers (3rd).docx [file pone.0276568.s006.docx]

**PONE-D-22-27071R2**

**Effects of a choral program combining wind instrument performance and breathing training on respiratory function, stress, and quality of life in adolescents: A randomized controlled trial**

**PLOS ONE**

Before responding to the review comments, thank you for your interest in our study.

***Reviewer 1***

Thanks a lot for your help in the revision of my thesis. May God bless you always...

***Reviewer 2***

Thanks a lot for your help in the revision of my thesis. May God bless you always...

***Reviewer 4***

**<Minor revisions>**

1) 1- Line 97: This sentence is poorly worded. "Through the screening process, 0 subjects were excluded as they dissatisfied the inclusion criteria or satisfied the exclusion criteria, and a total of 50 subjects participated in this study." Consider the following instead. "During the screening process, all subjects satisfied the inclusion and exclusion criteria. A total of 50 subjects participated in the study."

A: We have accepted your request and completed the correction

2) 2- Simplify line 101: The study was conducted from March to September 2020.

A: We have accepted your request and completed the correction

**With the above, we will send the responses to the reviewers' review opinions of this researcher.**

**I would like to thank this researcher for helping me to publish a better study, and I would appreciate it if you would always give me advice if there are any deficiencies in corrections.**

**P.s. God bless you always**
